# Ultrasound-assisted recovery of humic substances from municipal digestate using response surface methodology^☆^

**DOI:** 10.1016/j.ultsonch.2026.107863

**Published:** 2026-04-26

**Authors:** Shon George Shiju, Krishnakumar Chullipalliyalil, Brijesh K. Tiwari, Patrick Quille, Gaurav Rajauria

**Affiliations:** a School of Microbiology, School of Food and Nutritional Sciences, University College Cork, Cork, Ireland; b SUSFERM Centre for Sustainable Fermentation and Bioprocessing Systems for Food and the Bioeconomy, University College Cork, Cork, Ireland; c Centre for Advanced Photonics and Process Analysis, Munster Technological University, Cork, Ireland; d Department of Food Chemistry and Technology, Teagasc Food Research Centre, Dublin 15, Ireland; e Plant Biostimulant Group, The Centre for Applied Bioscience Research, Munster Technological University, Tralee, Co. Kerry, Ireland

**Keywords:** Anaerobically digested sludge, Humic acid, Fulvic acid, Digestate valorisation, Sustainable circular system, Urban waste

## Abstract

Anaerobic digestion (AD) is widely employed for sewage sludge stabilization, producing substantial volumes of digestate that require further management. This study explores the recovery of high-quality humic substances (HS), specifically humic acids (HA) and fulvic acids (FA), from anaerobically digested sewage sludge (DSS) using ultrasound-assisted extraction (UAE). Implementing this step prior to downstream digestate management positions DSS as a low-cost, sustainable feedstock to produce high-value agronomic biostimulants and soil conditioners. Response surface methodology (RSM) was applied to optimize the UAE parameters, namely ultrasound amplitude (20–100%) and extraction time (5–20 min), to maximise HA yield. A second-order polynomial model demonstrated a strong fit to the experimental data (R^2^ ≥ 0.90), achieving HA and FA yields of 424.67 ± 0.03mg g^−1^ and 419.04 ± 0.01 mg g^−1^ (dry basis), respectively. Under optimized conditions (97% amplitude, 8.4 min, 0.1 M KOH, 1:40 v/w), HA yields increased by 109% compared to conventional extraction (CE: 200 rpm, 16 h), with a concurrent 85.8% reduction in energy consumption (CE: 0.281 kWh vs. UAE: 0.03 – 0.04 kWh). Elemental profiling highlighted the superior quality of UAE-HAs, characterised by significant carbon intensification (44.64% vs 37.9%), nitrogen recovery (6.69% vs 0.74%) and sulphur enrichment (1.95% vs 0.07%) relative to the commercial standard. Spectrometric analysis further corroborated these findings, revealing greater functional group diversity in DSS-derived samples, positioning them as superior candidates for agricultural applications. These findings demonstrate the potential of UAE as an efficient and economical technology for HS recovery, advancing the circularity of digestate-based biorefineries by transforming urban organic waste streams into value-added agronomic resources.

## Graphical abstract

**Figure:**
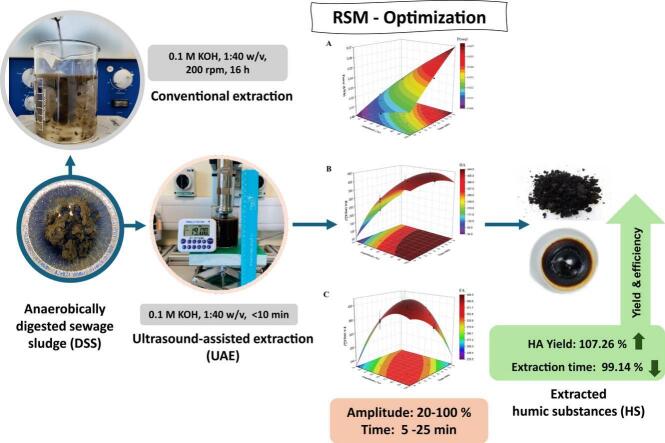

## 1 Introduction

In recent years, anaerobic digestion (AD) has emerged as a sustainable solution for effectively managing sludge from both industrial and municipal waste streams. Besides producing biogas, the AD process also creates a nutrient-rich byproduct called digestate. The estimates from the European Biogas Association (EBA) indicate that over 31 million tonnes (Mt) dry digestate was produced in 2022, with projections rising to 177 Mt year^−1^ by 2050 [1]. Over the years, the AD process has become a leading method for sludge management at wastewater treatment plants (WWTPs) [2]. In 2022, WWTPs across the European Union (EU) generated approximately 5.43 Mt of sludge (Table S1). However, only a small portion, about 0.05 Mt, underwent biostabilisation processes [3]. This highlights a significant untapped potential, particularly as AD adoption increases across Europe and outside. Even though AD reduces total solid levels (TS) and organic matter (OM) content (10–25% mass reduction), the produced digestate wet volumes are two- to three-fold than that of the feedstock in a wet-AD system [4]. Digestate management remains a significant barrier to the advancement of AD as a fully sustainable and economically efficient solution, particularly when utilising heterogeneous feeds such as municipal sludge [5], [6]. Above all sludge/digestate production is anticipated to increase as the population is growing, and treatment facilities are on the rise. Nutrient recycling through agricultural land spreading of digestates has been the prominent disposal route [6], [7].. Nevertheless, residual methane emissions, erratic fertilising potential, pathogenic and heavy metal contamination and regulatory oversight continue to constrain this traditional approach [2], [8].

Anaerobic biostabilisation processes have shown accelerated humification of OM and in-situ humic acid (HA) formation in the digestate, which at times serves as process maturity indicators. Sewage sludge has been shown to possess 14.6% humic substances (HS) of TS (27.0% of OM), with over 90.6% of it accounted by HA [9], compared to peat samples which consists of around 40% HS [10]. Digestates typically contain undigested inorganic and organic material alongside microbial biomass, minerals and HS. These structurally complex classes of HS are classified based on solubility into humic acids (HA, insoluble at pH < 2), fulvic acids (FA, soluble across all pH) and humin (insoluble residue). The HA and FA fractions make up the most researched fraction of extractable HS studied. Alongside solubility differences, HA are characterised by higher molecular weight, degree of polymerization, and increased acidic functional groups as with FAs [11].

Distinctive chemical structure, abundantly active redox functional groups, greater electron transfer capacity among other unique characteristics have established HS as a versatile class of compounds that finds applications ranging from agricultural (fertiliser, redox buffer, soil structure, soil microbiome promoter) [12], electrochemistry (detection sensor, energy storage) [13], environmental remediation (heavy metal conjugation, contaminant removal) [14], [15], plant science (biostimulant, disease resistance) [16], [17], [18], materials science (mineral interactions, binders and strengtheners) [19], wellness and pharmaceuticals [20]. According to Straits Research, the HS market was valued at approximately USD 0.61 billion in 2022 with a forecast reaching over 1.7 billion by 2031, which equates to circa 12% CAGR [21]. Primarily pushed by the agri sector; humic-based fertilisers are substituting synthetic mineral fertilisers. The report highlighted increased awareness and policy-direct encouragement of bio-based resources as additional drivers for the market growth, with Asia Pacific being the fastest and Europe the largest market. The ubiquitous, eco-friendly and relatively affordable nature further signifies high-value application targeting [13].

Low-rank coal (peat: 10–40% HS, leonardite and lignite: 10–30% HS) [22], [23], [24], [25], soil, and sea sediments (2–5% HA) [26], [27] are the predominant sources for HA extraction [25]. The conventional method described by Stevenson involves using a strong alkali to extract soluble materials like dissolved organic matter, followed by recovering HA through acidification [11]. Furthermore, Roger S. Swift's introduction of column adsorption to fractionate hydrophobic and hydrophilic FA was endorsed by the International Humic Substances Society (IHSS) and is routinely accepted by researchers while extracting soil HS [28]. The ability of high pH and elevated ionic strength to enhance abstraction of hydrophilic fractions reinforces the prominence of the alkaline extraction method [29]. These methods are unified by their alkaline extractant and prolonged mechanical agitation, sometimes as long as 24 h, leading to low extraction efficiencies [23]. To overcome low extraction kinetics, various high-energy methods like ultrasound, microwave and hydrothermal technologies have been explored [30], [31]. Ultrasound application has been explored as a pretreatment for various AD feedstock preparations, dissolved organic matter (DOM) extraction [32], post-treatment sludge management, and HS recovery [23], [27], [33], [34]. The implementation has significantly reduced process times while simultaneously intensifying mass transfer in comparison to conventional extraction (CE) methods.

**Fig. 1:**
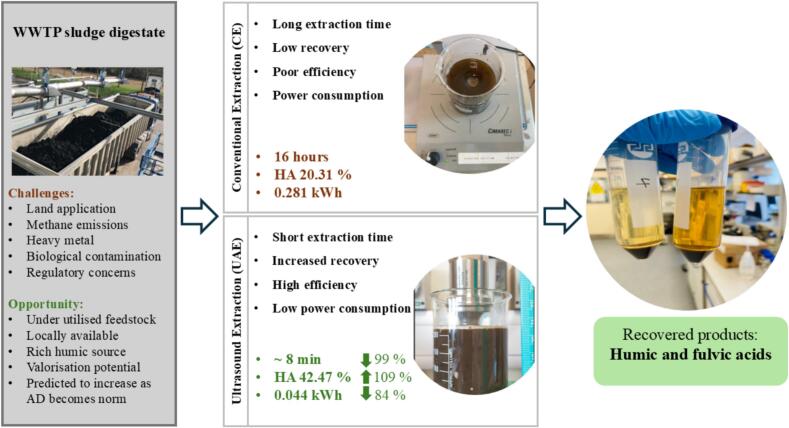
Schematic comparison of conventional extraction (CE) and ultrasound-assisted extraction (UAE) for recovering humic and fulvic acids from underutilised digestate from the wastewater treatment plant (WWTP).

Despite the growing interest in HA and advancements in extraction techniques, much of the research remains concentrated on competitive yet fossil-based substrates such as peat and lignite. However, sludge digestates, a widely available and underutilized feedstock, have garnered increasing attention for their potential in value addition, aligning with circular economy principles and end-of-waste frameworks [2]. Utilizing biowaste residues as renewable feedstock not only supports ecosystem balance but also reinforces the waste-to-value approach embraced globally. In this context, the present study seeks to improve and optimize HS recovery by ultrasound-assisted extraction (UAE) from mixed municipal sludge digestates through response surface methodology (RSM). Fig. 1 outlines the challenges and untapped opportunities, while summarizing the main findings including a comparison between CE and optimized UAE pathways for HS recovery. To the authors' knowledge, no previous studies have specifically explored optimized HS recovery from digested sewage sludge (DSS). Therefore, this research looks beyond the gaps in literature, offering key insights into digestate valorisation, enhancing resource recovery from organic waste streams, further supporting sustainable waste management practices and circular practices.

## 2 Materials and methods

### 2.1 Materials

Dewatered digestate (anaerobically digested sewage sludge) was obtained from a municipal WWTP in Co. Limerick, Ireland. Proximate analysis of digestate sample was carried out within 48 h, while the rest were stored in airtight plastic pails at 4 °C until further analysis.

### 2.2 Chemicals

Humic acid standard (1415–93-6), hydrochloric acid, and potassium hydroxide, were obtained from Sigma-Aldrich (Wicklow, Ireland). Chemical oxygen demand (COD) HR Plus TNT vials (0–15,000 mg L^−1^) were purchased from Reagecon (Clare, Ireland). Pierce™ Bradford Protein Assay Kit was sourced from Fisher Scientific (Ireland). All chemicals used, except the humic acid standard (technical grade), were of analytical grade, and Type II ultrapure water was used in extraction and analysis.

### 2.3 Feedstock characterisation

The pH (EN ISO 10390–2022), electrical conductivity (EC) (CEN TS 15937:2013), moisture (EN 15934:2012) and OM content (EN15935-2021) were determined by standard protocols. COD was estimated by the reactor digestion method [35] using Reagecon HR Plus TNT vials (product number: 420722) to manufacture's instructions (Method 800, Hach, Loveland, USA) while the elemental composition (CHNS) was obtained using a CNS928 and CHN828 series LECO elemental analysers (Laboratory Equipment Corporation, Michigan USA). The soluble protein was measured using a Pierce™ Bradford plus protein assay kit according to manufacturer instructions (product number: 23238).

### 2.4 Conventional extraction (CE)

The CE of extractable HS was carried out based on a modified protocol by Lamar et al., [36]. Briefly, a 5 g dry matter equivalent sample was mixed with 0.1 M KOH at 200 rpm at a 1:40 (w/v) solid-to-liquid ratio. The extraction of HS was carried out by means of mechanical agitation (200 rpm) overnight at room temperature, with the headspace purged and sealed under N_2._ The obtained crude extract containing DOM was centrifuged at 12,000 × g for 20 min and a supernatant containing both HA and FA was collected. The HA was separated by acid precipitation, adjusting pH to 1.5 ± 0.2 using 6 M HCl, followed by centrifugation at 12,000 × g for 20 min, while the supernatant containing soluble FA was collected for recovery. Yields were estimated using thermogravimetric analysis (105 ± 10 °C until constant weight) and are expressed as milligram per gram of dry feedstock (mg g^−1^_db_).

### 2.5 Ultrasound-assisted extraction (UAE)

Ultrasound studies were carried out using 5 g dry matter equivalent feedstock, under 1:40 (w/v) 0.1 M KOH extraction. The sludge digestates were disintegrated by mixing for 10 min at 200 rpm prior to ultrasonication using the pilot testing UIP 500 hdT sonicator (20 kHz, 500 W, Hielscher, Germany) operating at 20  kHz with an 18  mm diameter probe (BS4d18). Details on the process variables such as time and amplitude tested are described in section 2.6. The extracts obtained were processed as described for CE.

The ultrasound power (P), intensity (UI), and power density (PD) were determined using Eq. (1), 2 and 3, as described in [37] respectively.

(1) $$Ultrasound\;Power\;\left(P\right)=m{.C}_{p}.(dT/dt)$$

P being the power in watt (W); m the solvent mass (g); Cp being the solvent heat capacity at constant pressure (J.g^−1^.℃^−1^) and dT/dt the temperature change per second (℃.s^−1^).

(2) $$Ultrasonic\;intensity\;\left(UI\right)=\frac{4.P}{\pi {.D}^{2}}$$

UI is the ultrasonic intensity (W.cm^−2^); P being power in watts (W); and S is the transducer emitting surface area (cm^2^).

(3) $$Ultrasonic\;power\;density\;\left(PD\right)=\frac{P}{V}$$

PD being Power density (W.cm^−3^ or W.L^−1^); P is the power in watts (W); and V the total solution volume in cubic centimetre (cm^−3^).

### 2.6 Experimental design and statistical analysis

A five-level, 2 factor, central composite design (CCD) used in this study was designed using Minitab® 19 (v 19.1). The coded (−α, −1, 0, 1, +α) and actual values for the independent variables selected are listed in Table 1, with √2 being the α value used in this experimental design.

**Table 1:** Independent variables and their coded levels used for ultrasound-assisted extraction (UAE) optimisation of humic substances (HS) extraction from digested sewage sludge (DSS).

Independent parameters (units)	Tag	Symbol	Parameter levels				
			−α	–1	0	1	+α
US amplitude (%)	A	X_1_	20	40	60	80	100
Extraction time (min)	B	X_2_	5	10	15	20	25

A total of 13 runs, with 5 centre-point replicates for optimization of ultrasonic amplitude (20–100%) and treatment time (5–25 min) were carried out. Extraction yields for HA (mg g^−1^_db_), FA (mg g^−1^_db_), and power consumed (kWh) from the CCD were analysed using response surface regression (Table 4) using Minitab® 19 (v. 19.1; Minitab LLC, USA) and fitted to a second-order polynomial model (Eq. (4)).

(4) $$Y=\;\beta_{0}+\;\sum_{i=1}^{k}\beta_{i}X_{i}+\;\sum_{i=1}^{k}\beta_{ii}X_{i}^{2}\;+\;\;\sum_{i}^{k-1}\sum_{j}^{k}\beta_{ij}X_{i}X_{j}$$

Where, Y is the predicted response (power, HA, or FA yield); $\beta_{0}$ is the constant coefficient; $\beta_{i}$ is the linear coefficient; $\beta_{ii}$ is the quadratic coefficient; $\beta_{ij}$ is the cross-product coefficients $X_{i}$ and $X_{j}$ are the two tested independent variables.

A three-dimensional colormap surface with projections was generated using the 3D function plot tool in OriginPro® (v.10.1.5.132, 2024b; OriginLab Inc., USA), while keeping other variables constant in the second-order polynomial model. Model validity was assessed by comparing experimental and predicted values at 5% significance levels (α = 0.05).

### 2.7 Humic substances analysis

The isolated HSs were analysed using spectrometric techniques to assess their compositional and structural characteristics, and the results were benchmarked against reference humic acid sodium salts obtained from Sigma-Aldrich.

#### 2.7.1 Elemental analysis

The elemental composition of extracted HS was determined using CNS928 and CHN828 series LECO analysers (LECO Corporation, Michigan, USA). The samples were dried at 105 ± 1 ˚C overnight to minimise moisture and tested in triplicates in accordance with manufactures recommendations. The percent oxygen content was calculated by difference.

#### 2.7.2 UV–Vis spectroscopy

UV spectral scans (190–1100 nm, 1 nm increments) of HS samples were recorded with a GENESYS UV–Vis spectrophotometer (ThermoFisher Scientific, Dublin, Ireland) using 0.2 mg ml^−1^ HS dissolved in 0.02 M KOH. The measurements were carried out on 0.45 µm PVDF membrane-filtered samples at 20 °C, using 0.02 M KOH as blank in quartz cuvettes [38]. The values were normalised and baseline transformed prior to plotting in OriginPro® (v 10.1.5.132, 2024b; OriginLab Inc., USA).

#### 2.7.3 Fourier-transform infrared spectroscopy (FTIR)

The powdered HA and FA obtained from CE and UAE optimized conditions were pre-dried at 60 ± 2 ˚C for 20 min and cooled to room temperature, in a desiccator prior to obtaining the FTIR spectra at 500 – 4000 cm ^−1^ using PerkinElmer Spectrum 400 FTIR/NIR imaging system (PerkinElmer, Buckinghamshire, UK) fitted with universal attenuated total reflectance (UATR) accessory. The readings were obtained with a resolution of 4 cm^−1^ over 16 scans, and the absolute values were used to generate the spectral plot in OriginPro® (v 10.1.5.132, 2024b; OriginLab Inc., USA) with individual Y-axis offset.

#### 2.7.4 Scanning electron microscopy (SEM) analysis

The surface morphology of feedstock, and extraction residues were analysed using an S-3700 N variable pressure scanning electron microscope (SEM) (HITACHI, Tokyo, Japan). Oven dried (60 ± 2 ˚C) DSS, and residues from CE and optimized UAE were imaged over carbon tape under 30 Pa (0.225 Torr) vacuum, 15 keV, and 162.0 µA, while observing for secondary electrons [39].

### 2.8 Energy and preliminary economic assessment

Energy consumption during CE and UAE was measured using a Brennenstuhl energy monitor (model 1506603, Tübingen, Germany). Energy intensity (EI) was calculated as the ratio of electrical energy consumed to the mass of HA recovered per extraction run (kWh g^−1^ HA) (Eq. (5), and the chemical intensity (CI) was calculated the total mass of reagents added per unit mass of HA, as shown in Eq. (6).

(5) $${\mathrm{Energy}\;\mathrm{inte}ns{\mathrm{ity}}_{x}\;(EI}_{x})={\left(\frac{E}{Y_{\mathrm{HA}}}\right)}_{x}$$

*where* $EI_{x}$ *is the energy intensity of extraction process* x *(kWh g^−1^ HA); E is the measured electrical energy consumption (kWh); and* $Y_{\mathrm{HA}}$ *is the mass of humic acid recovered (g);* x *denotes the extraction method, conventional extraction (CE) or ultrasound-assisted extraction (UAE).*

(6) $${\mathrm{Chemical}\;\mathrm{inte}ns{\mathrm{ity}}_{r,x}\;(CI}_{r,x})=\;{\left(\frac{Y_{r}}{Y_{\mathrm{HA}}}\right)}_{x}$$

*where* $CI_{r,x}$ *is the chemical intensity of reagent r in extraction process* x *(g reagent g^−1^ HA);* $Y_{r}$ *are the mass of reagent r (potassium hydroxide or hydrochloric acid) added during the extraction x (g);* $Y_{\mathrm{HA}}$ *is the mass of humic acid recovered (g);* x *denotes the extraction method, conventional extraction (CE) or ultrasound-assisted extraction (UAE).*

A preliminary operating cost (OC) assessment was conducted to compare CE and UAE based on direct, variable inputs, namely electrical energy and chemical reagents. Energy costs were calculated using the measured energy intensity and an average European industrial electricity price (€ kWh^−1^), as reported by Eurostat [40] (Eq. (7)). Chemical costs were estimated from reagent intensities and current laboratory-scale reagent prices (Sigma-Aldrich), expressed as pure chemical equivalents (Eq. (8)). Capital expenditure, labour, maintenance, waste handling, reagent recovery, and scale-dependent efficiencies were excluded from the analysis.

(7) $$C_{\mathrm{energy},x}={\mathrm{EI}}_{x}\times p_{e}\times 1000$$

*where* $C_{\mathrm{energy},x}$ *is the energy cost (€ kg^−1^HA) for extraction process; x,* ${\mathrm{EI}}_{x}$ *is the energy intensity (kWh g^−1^H); and* $p_{e}$ *is the average 2025 European electricity price (0.1902 € kWh^−1^);* x *denotes the extraction method, conventional extraction (CE) or ultrasound-assisted extraction (UAE).*

(8) $$C_{\mathrm{chem},x}=\sum_{r}{\left({\mathrm{CI}}_{r}\times p_{r}\right)}_{x}\times 1000$$

*where* $C_{\mathrm{chem},x}$ *is the total reagent (KOH and HCl) cost (€ kg^−1^ HA) for extraction x;* ${\mathrm{CI}}_{r}$ *the chemical intensity of reagent r (g reagent g^−1^ HA); and* $p_{r}$ *is the unit reagent price from (€ g^−1^));* x *denotes the extraction method, conventional extraction (CE) or ultrasound-assisted extraction (UAE).*

The total preliminary operating cost was calculated as the sum of energy and chemical costs normalised to humic acid recovery, yielding an indicative cost of production (€ kg^−1^ HA), as shown in Equation (9). Representative energy and chemical cost calculations are provided in the Table S2.

(9) $$Operating\;cost\;\left({\mathrm{OC}}_{x}\right)={\left(C_{\mathrm{energy}}+C_{\mathrm{chem}}\right)}_{x}\;\;$$

*where O* $C_{x}$ *is operating cost (€ kg^−1^ HA) for extraction x,* $C_{\mathrm{energy},x}$ *is the energy cost (€ kg^−1^ HA);and* $C_{\mathrm{chem},x}$ *is the total reagent (KOH and HCl) cost (€ kg^−1^ HA);* x *denotes the extraction method, conventional extraction (CE) or ultrasound-assisted extraction (UAE).*

## 3 Results and Discussion

All results are expressed on dry matter basis, determined in experimental runs, providing accurate and reliable result representations.

### 3.1 Feedstock characteristics

The DSS, sourced from municipal WWTP were anaerobically digested and mechanically dewatered before collection. The proximate analysis was carried out on oven dried **(**60 ± 5 ˚C) DSS and are presented in Table 2. The mechanically dewatered DSS had relatively high moisture content (80.5%) upon receiving, with elevated ash levels (35.56%). These are normal for sewage sludge-based feeds [41]. The DSS, with its high OM (64.44%) presents itself as a promising feedstock for cleaner HS extraction, comparable to conventionally used carbonaceous materials like brown coal, lignite, leonardite, peat etc [42], [43], [44]. The elemental analysis shows one third of the composition is made up of carbon and nearly 5% with nitrogen.

**Table 2:** Characteristics of digested sewage sludge (DSS).

Parameters	Value ± S.D.	Unit
pH	8.64 ± 0.01	
E.C	1032 ± 1.73	µS cm^−1^
Moisture (%)	80.05 ± 0.04	%
Dry matter (%)	19.95 ± 0.04	%
Organic matter (%)	64.44 ± 0.63	% db
Ash (%)	35.56 ± 0.63	% db
COD	13890.67 ± 10.97	mg L^−1^ COD
Total carbon (C)	33.73 ± 0.06	% db
Total hydrogen (H)	4.72 ± 0.08	% db
Total nitrogen (N)	4.95 ± 0.02	% db
Total sulphur (S)	1.64 ± 0.03	% db
Oxygen (O) ^a^	57.3 ± 0.3	% db
Soluble protein	8.01 ± 1.91	g kg^−1^ db

E.C: Electric conductivity; COD: Chemical oxygen demand; S.D.: Standard deviation; mS: milliSiemens; db: dry basis; ^a^ Calculated by difference.

### 3.2 Conventional extraction

The extraction efficiency for HSs (HA and FA) was calculated as mg per g feedstock, over a dry matter basis (mg g^−1^_db_). The results are presented in Table 3. The crude extract of FA was defined as the total isolate obtained after the precipitation of HA, leading to 20.31% HA and 43.31% FA yields over a 16-hour extraction. The fulvic yields were sizable over reported studies, while HA were in line with other conventional feedstock and extraction methods. Fatima et al. (2021) obtained roughly 21.15% and 11.6% HA yields from lignite and bituminous coal respectively, when using alkaline extractions [45]. Jarukas et al. (2021) reported HA and FA values to be between 2 and 15.3% and 0.25 – 1%, respectively when comparing between black, brown and light peat as feedstocks [46]. The authors also found active maceration to have higher active organic compounds over UAE. When it comes to raw sewage sludge, Gayathri et al. (2020) obtained 4.87% HA and 2.75% FA recovery [47].

**Table 3:** Humic substance (HS) yields (± σ) obtained over conventional extraction (CE).

Sl. no	Method	Extraction time (min)	Power(kWh)	HA ± S.D. (mg g^−1^_db_)	FA ± S.D. (mg g^−1^_db_)
1	CE	960	0.281	203.16 ± 0.007	434.16 ± 0.03

CE: Conventional extraction; HA: Humic acid; FA: Fulvic acid; S.D.: Standard deviation.

### 3.3 Modelling ultrasound-assisted extraction

The response dependant variables (power, HA, and FA) for each experiment in the design space are listed in Table 4. The yields varied from 149.76 to 444.00 and 311.76 to 426.18 mg g^−1^_db_ for HA and FA respectively.

**Table 4:** Experimental design matrix and their responses.

Sl. no	US amplitude (%)	Extraction time (min)	PD (W cm^3^)	pH	Power (kWh)	HA (mg g^−1^_db_)	FA (mg g^−1^_db_)
1	60	25	0.128	12.42	0.048	424.04	375.80
2	80	20	0.174	12.25	0.048	417.36	359.22
3	40	10	0.134	12.93	0.016	326.26	387.16
4	60	15	0.194	12.37	0.031	397.92	410.74
5	100	15	0.270	12.15	0.045	444.00	364.84
6	60	5	0.286	12.31	0.011	313.62	366.62
7	60	15	0.198	12.35	0.031	376.66	403.74
8	60	15	0.194	12.28	0.031	398.90	418.88
9	20	15	0.046	13.07	0.013	149.76	311.76
10	80	10	0.303	12.35	0.027	424.06	397.56
11	60	15	0.190	12.36	0.031	397.34	418.04
12	40	20	0.120	12.76	0.031	373.62	426.18
13	60	15	0.190	12.76	0.031	429.64	375.48

US: Ultrasound; PD: Power density; HA: Humic acid; FA: Fulvic acids.

The effect of experimental factors (US amplitude and extraction time) was quantified by fitting the data to a second-order polynomial equation (Eq. (4)). Table 5 and Table 6 presents the analysis of variance (ANOVA) of the regression parameters of the predicted RSM. The effect of linear, quadratic and cross products in the model was adequacy tested to describe the response surfaces. The model presented showed high correlation coefficients (R^2^ of 1.0 (power), 0.99 (HA) and 0.70 (FA)) along with low coefficients of variation (CV < 8.8%) (Table 5). Response surface ANOVA showed both linear and quadratic models to be significant (p < 0.05) for power and HA, while cross products or interaction among the parameters were insignificant unless for power (p < 0.01). Therefore, power and HA yields were only influenced strongly by independent variables linearly and quadratically.

**Table 5:** Analysis of variance (ANOVA) table showing effect of independent variables on dependent variables as linear, quadratic and interactive terms.

Term	Power (kWh)	HA (mg g^−1^_db_)	FA (mg g^−1^_db_)
Linear (p)	0.0000^⁎⁎⁎⁎^	0.001^***^	0.807^ns^
Quadratic (p)	0.018^⁎^	0.019^**^	0.028*
Cross product (p)	0.003^**^	0.452^ns^	0.137^ns^
Lack of fit (p)	^nc^ (pure error = 0)^a^	0.071	0.192
Total model (p)	< 0.0001	0.002^**^	0.084^ns^
RMSE	0.00068	0.03188	0.02303
CV	2.45	8.77	5.99
R^2^	0.9981	0.9023	0.6920

* Significant at p ⩽ 0.05; ^**^ Significant at p ⩽ 0.01; ^***^ Significant at p ⩽ 0.001; ^****^ Significant at p ⩽ 0.00001; ^nc^ not calculated; ^ns^ not significant; ^a^ Lack-of-fit test not valid due to zero pure error, HA: Humic acid; FA: Fulvic acid, RSME: Root mean square error; CV: coefficient of variation..

**Table 6:** Regression coefficients and ANOVA of regression parameters of the predicted response surface quadratic models.

Coefficient	Power	HA	FA
Intercept			
β0	−0.0136600^***^	−0.261^****^	−0.006^****^
Linear			
β1	0.0003060^****^	0.01271^****^	0.00807^ns^
β2	0.0013760^****^	0.0215*	0.02191^ns^
Quadratic			
β11	−0.0000010^**^	−0.000064^**^	−0.000041*
β22	−0.0000015*	−0.0003^ns^	−0.000333^ns^
Cross product			
β12	0.0000150^**^	−0.000135^ns^	−0.000193^ns^
p value	<0.0001^****^	0.002^**^	0.084^ns^

β0 is the constant coefficient; β1 and β2 are the linear coefficient for US amplitude and extraction time respectively; β11 and β22 are the quadratic coefficient for US amplitude and extraction time respectively; β12 is the cross-product coefficients; * Significant at p⩽0.05; ^**^ Significant at p⩽0.01; ^***^ Significant at p⩽0.001; ^****^ Significant at p⩽0.00001; ^ns^ not significant; HA: Humic acid; FA: Fulvic acid.

**Fig. 2:**
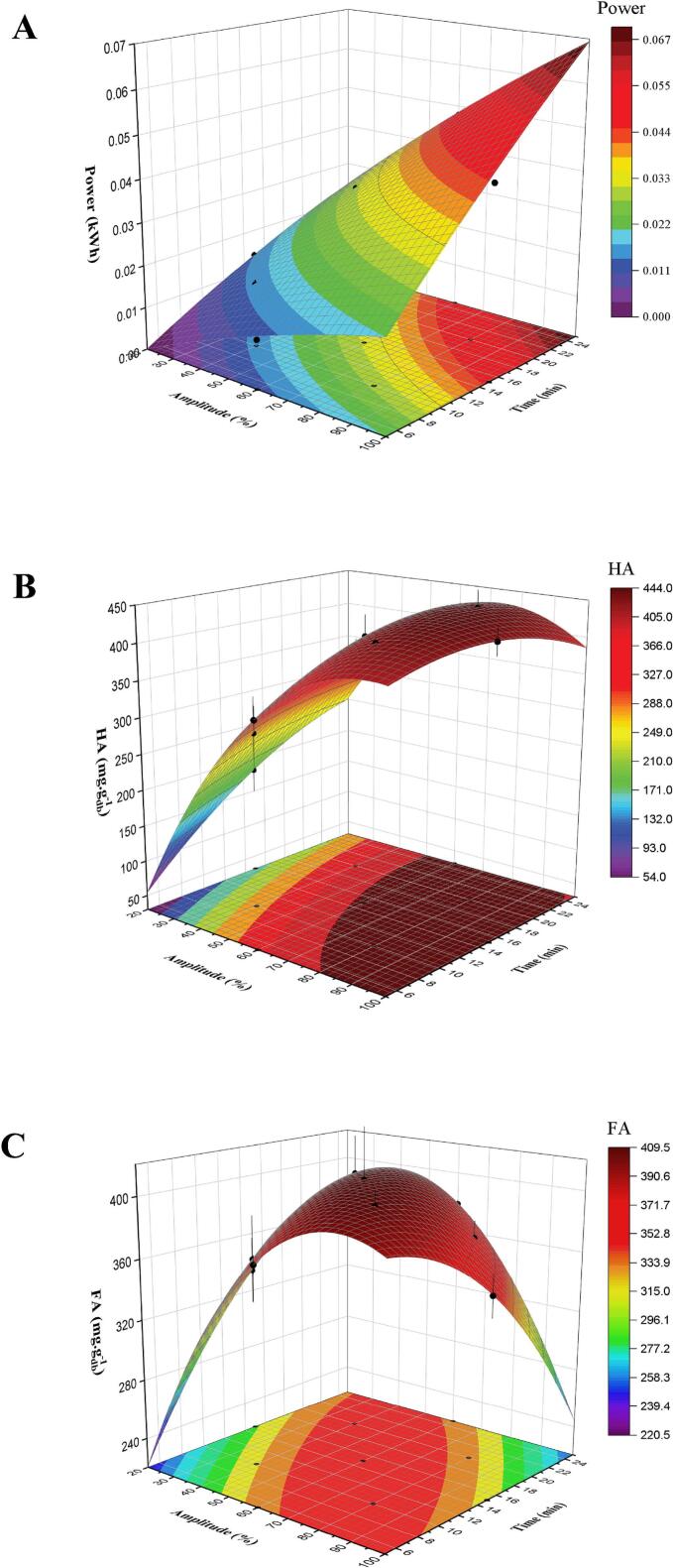
3D surface plots of (A) power, (B) HA yield, and (C) FA yield as a function of amplitude and extraction time using ultrasound-assisted extraction (UAE) from digested sewage sludge (DSS).

Adequate model fit is a much required for avoiding ambiguous results [48]. Eq. (10), (11), and (12) describes the relationship of US amplitude and extraction time (independent variables), to the response variables, power, HA, and FA, respectively. Response surface plots illustrate the relationship (power (Fig. 2.A), HA (Fig. 2.B), and FA (Fig. 2.C)) graphically as function of modelled parameters.

Relationship between power and independent variables

(10) $$Power\;\left(kWh\right)=\;-0.01366\;+\;0.000306\;X\;+\;0.001376\;Y\;-\;0.000001\;X^{2}\;-\;0.000015\;Y^{2}\;+\;0.000015\;XY$$

Relationship between HA and independent variable

(11) $$HA\;\left(mg{\;g}_{\;\;\;\;db}^{-1}\right)=\;-0.261+0.01271\;X\;+\;0.0215\;Y\;-\;0.0000637\;X^{2}\;-\;0.000300{\;Y}^{2}\;-\;0.000135\;XY\;$$

Relationship between FA and independent variable

(12) $$FA\;\left(mg{\;g}_{\;\;\;\;db}^{-1}\right)=\;\;-0.006+0.00807X\;+\;0.02191\;Y\;-\;0.0000414\;X^{2}\;-\;0.000333\;Y^{2}\;-\;0.000193\;XY$$

### 3.4 Optimisation of ultrasound-assisted extraction

Two solutions were obtained, maximising HA and maximizing both HA and FA. The predicted along with experimental validation values are given in Table 7. A high degree of desirability values (0.96 and 0.89) indicated strong confidence in the model and prediction. The validation values were 424.7 mg g^−1^_db_ and 399.4 mg g^−1^_db_ HA within negligible deviation when comparing with predicted values. Whereas for maximising HA, and FA content, the values were similarly accurate, with percent errors of 0.02 and 0.28 for HA and FA, respectively. Overall, the experimental results closely matched these predictions, with absolute errors ranging from 1.16 to 3.27 mg g^−1^_db_ and percent errors under 0.8% for both products recovered, underscoring the reliability and precision of the model.

**Table 7:** Experimental vs. predicted values from response surface methodology (RSM) optimised ultrasound-assisted extraction (UAE).

Terms		US amplitude	Time	Power	HA ± S.D.	FA ± S.D.	Desirability
		(%)	(min)	(kWh)	(mg g^−1^_db_)	(mg g^−1^_db_)	
a.	Maximize HA (predicted value)	97.576	8.434	0.044	421.39	NA	0.961
Experimental value				0.044	424.67 ± 0.03	430.09 ± 0.02	
absolute error				0	3.275	NA	
percent error				0	0.771	NA	
b.	Maximize HA, FA (predicted value)	91.111	7.02	0.027	399.47	417.89	0.887
Experimental value				0.027	399.40 ± 0.19	419.04 ± 0.01	
absolute error				0	0	1.156	
percent error				0	−0.018	0.276	

US: Ultrasound; HA: Humic acid; FA: Fulvic acid; S.D.: Standard deviation; NA: Not applicable.

Optimised UAE significantly enhanced HA yields with respect to CE, increasing from 203.16 to 424.67 mg g^−1^_db_ (109%), while FA values remained nearly unchanged (434.16 vs. 430.09 mg g^−1^_db_), all at a substantially lowering power consumption (CAE: 0.281 vs. UAE: 0.03–0.04 kWh) and duration (12–16 h vs. < 10 min). Similar observations, of HA doubling (22.60% to 54.51% HA_daf_) were seen when Nieweś et al., used a 45 kHz sonication bath at 400 mW cm^−2^ for 45 min. Efficiency up to 60.28% HA was obtained when treated for 135 min at 400 mW cm^−2^ [49]. Long-term sonication has been shown to negatively affect HA yield, as they disintegrate into lower molecular mass products [45], [49]. This was not evident here, likely due to the lower treatment time of less than 30 min. Mecozzi et al. experimenting with ultrasound coupled alkaline extraction of HS from marine sediments found the presence of no additional degradation products when compared to non-sonicated samples [27]. A recent study looking at RSM optimisation was able to achieve HA recovery as high as 89.32% from coal samples [50].

### 3.5 Characterization of extracted HA

#### 3.5.1 Elemental analysis

The elemental composition and oxygen content of the extracted HS are summarized in Table 8. The digestate derived HAs exhibited a distinct elemental profile compared to the commercial standard. Notably, the implantation of UAE facilitated carbon intensification, yielding the highest carbon content (44.64%), which surpassed both CE (41.2%) and standard HA (37.88%). This trend aligns with previous findings indicating that HS derived from organic waste streams typically possess higher organic carbon and nitrogen loads compared to soil or coal-based [50], [51], [52], [53]. Another critical differentiation was the substantial nitrogen enrichment. While the standard HA contained negligible nitrogen (0.74%), the extracted samples exhibited levels ranging from 6.48% (CE) to 6.69% (UAE). This elevated N content is attributed to the protein-rich nature of microbially digested feedstocks. Additionally, the alkaline extraction and HS recovery likely favoured the co-precipitation of protein-humic fractions due to their solubility characteristics [54], [55].

**Table 8:** Elemental composition (CHNSO) of humic substances (HS) extracted from digested sewage sludge (DSS) via conventional (CE) and ultrasound-assisted (UAE) methods.

Sample		Carbon (C)	Hydrogen (H)	Nitrogen (N)	Sulphur (S)	Oxygen ^a^ (O)
		mean ± S.D (% db)				
Standard	HA	37.88 ± 0.12	2.27 ± 0.09	0.74 ± 0.03	0.07 ± 0.01	65.2 ± 0.16
CE	HA	41.21 ± 0.92	6.31 ± 0.09	6.48 ± 0.07	1.41 ± 0.02	43.59 ± 1.30
	FA	8.09 ± 0.19	2.63 ± 0.10	3.80 ± 0.22	0.73 ± 0.01	84.75 ± 0.32
	R	28.36 ± 1.11	4.45 ± 0.09	2.89 ± 0.15	0.15 ± 0.04	64.15 ± 0.85
UAE	HA	44.64 ± 0.24	5.95 ± 0.07	6.69 ± 0.11	1.95 ± 0.00	40.77 ± 0.26
	FA	5.34 ± 0.25	3.13 ± 0.08	1.83 ± 0.12	0.08 ± 0.01	89.62 ± 0.28
	R	21.49 ± 0.88	4.88 ± 0.09	1.85 ± 0.04	0.39 ± 0.06	71.4 ± 0.66

db: dry basis; S.D.: Standard deviation; CE: Conventional extraction; UAE: Ultrasound-assisted extraction; HA: Humic acid; FA: Fulvic acids; R: Residue; calculated by difference.

From a fertiliser product perspective, this elemental profile positions digestate-derived HS as a superior resource for agricultural applications compared to lignite-derived standards [16], [18]. Conversely, the FA fractions displayed significantly lower carbon and nitrogen concentrations, reflecting a lower degree of humification and maturity. This distribution agrees with previous reports on municipal solid waste fractionation, where humic-like structures preferentially accumulate in the HA fraction [51]. Overall, the elemental composition of the UAE-recovered HS is consistent with other organic waste extractions [51], [52], [55],validating this process as an efficient route for nutrient-dense HS recovery.

#### 3.5.2 UV–Vis spectrometric analysis

**Fig. 3:**
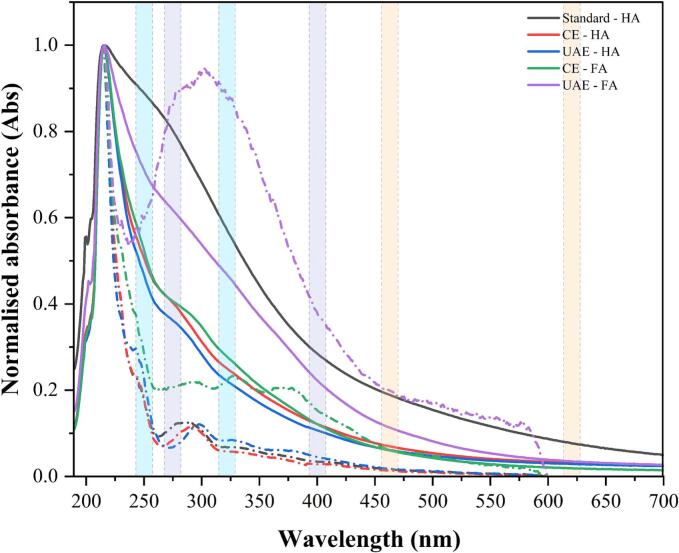
Normalised UV–Vis absorption spectra of humic substances (HA and FA) obtained from digested sewage sludge (DSS) via conventional extraction (CE) and ultrasound-assisted extraction (UAE).

**Fig. 4:**
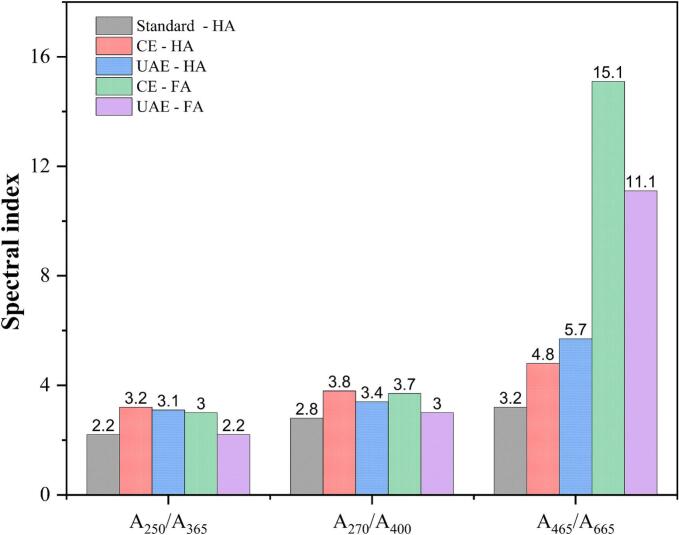
UV–Vis spectroscopic optical descriptors calculated for humic acid (HA) and fulvic acid (FA) obtained from digested sewage sludge (DSS) via conventional extraction (CE) and ultrasound-assisted extraction (UAE).

HSs exhibit a bonding affinity, often with metal ions, dictating the behaviour of the products. Their composition, frequently influenced by the binding of wastewater co-pollutants, strongly impacts this behaviour [39]. UV–Vis absorption spectral curves are impacted by the relative abundance of aromatic (−C=C-, 200–300 nm), ketonic (C=O, 180–290 nm), and other chromophoric functional groups (nitro groups (NO_2_), amines (NH_2_), and alkenes (C=C)). Auxochromes like amine (–NH_2_) and hydroxyl (–OH) groups modify electron transitions, affecting absorbance characteristics [56]. Fig. 3 shows the normalised UV–Vis absorption spectrum for standard HA, and both CE and UAE extracted HSs measured against 0.02 M KOH at 20℃. The dotted line indicates adaptive baseline transformation (coarseness – 10% and offset – 0) from 200 nm to 600 nm for respective absorption using SpectraGryph (v 1.2.17). Shaded bands are indicative for the wavelength regions used for abundance ratio calculation presented in Fig. 4. Absolute values were used for spectral index calculations.

Aligning with previous findings, the extracted HA was characterized by a gradually declining single absorption maxima in the region 200–290 nm [22], [39], [57], suggesting π → π* electronic transitions in aromatic systems. This results from overlapping absorbance of various chromophores in the humic core, including phenolic arenes, benzoic acids, aniline derivatives, polyenes, and polycyclic aromatic hydrocarbons with multiple rings [39], [58]. Niewes et al. (2022) have pointed out that the 280 nm local absorbance peak signifies the abundance of C=O chromophores characteristic of the hydrophobic fulvic fraction [22], which are present here as faint shoulder peaks in the extracts.

Literature studies have associated various absorption wavelengths (250, 254, 270, 280, 300, 365, 400, 465) and ratio (A_250_/A_365_, is A_270_/A_400_, A_465_/A_665_) for spectral differentiation, aromaticity and molecular weight determination, predominantly with good predictive capacities [44], [58], [59]. Fig. 4 shows the wavelength-specific abundance ratios of HSs extracted from DSS, indicating their humification degree, polymerisation, molecular weight and aromaticity. Absorption at shorter wavelengths near 270 nm is linked to the chromophore cores, while those from longer wavelengths are associated with carboxylated and aliphatic components possessing weaker chromophoric activity. The A_250_/A_365_ ratio evaluates molecular weight and aromaticity inversely, while A_270_/A_400_ gives insight into phenolic/quinoid core (chromophores) to carboxylic component proportions [44], [60], [61], [62]. UV absorptivity in the 270–280 nm range was used as an indicator of total aromaticity, corresponding to the π–π* electron transitions in multi-ring aromatic structures such as aniline derivatives, benzoic acids, phenolic arenes, polyenes, and other polycyclic aromatic hydrocarbons. Many of these compounds are considered characteristic of humic matter. Corelation between aromaticity and absorbance ration at 250 and 365 nm has been reported previously [58], [63]. Another widely used humification degree indicator is A_465_/A_665_, while also inversely indicative of condensation degree and molecular weights [64], where an index value less than 5 is regarded as a high degree of condensation [25]. In this case, aromaticity was slightly lower for extracted HA when compared to the standard. Variations in feedstock material could potentially be a reason for the complexity of HSs extracted. The FA show the existence of relatively larger aliphatic structures and lower aromatic condensation, especially when looking at A_464_/A_665_ from CE (A_464_/A_665_ − 15.1) which was higher than that of UAE (A_464_/A_665_ − 11.1). Kim et al (2006), ranked high-quality FA to have A_465_/A_665_ greater than 5 [65], with a general range falling between 10–13.1 [44]. Additionally, the extraction method appears to not only influence the bulk and aromaticity of HA but also lead to an increased complexion in the fulvic component, as seen with UAE. High degradation degree and chromophore content were seen in CE fulvic fraction while not affecting extracted HA. The resemblance in spectral fingerprint illustrates similarities in humic acids extracted by conventional and pretreated methods.

#### 3.5.3 Fourier transform infrared spectroscopy analysis

**Fig. 5:**
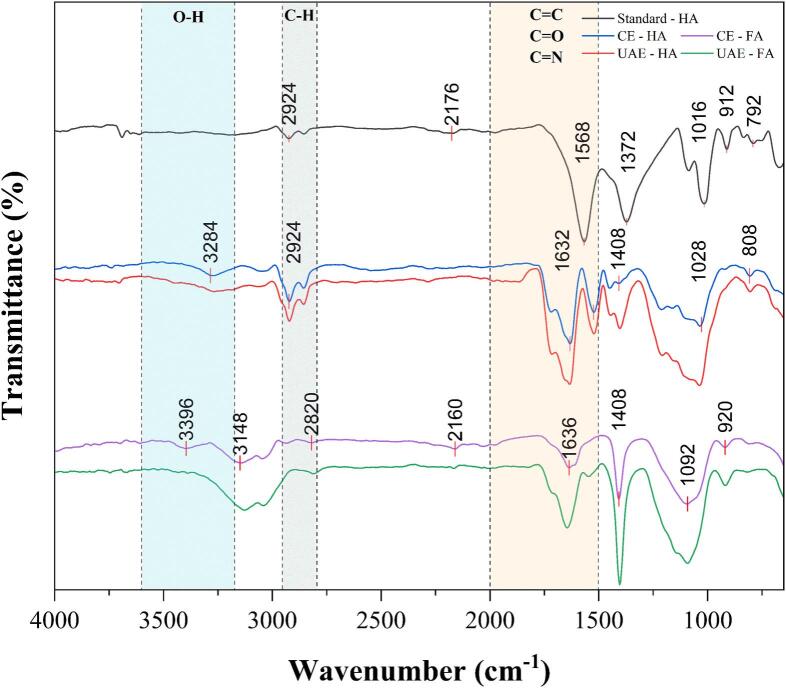
FTIR spectra for Humic substances (HA and FA) obtained from digested sewage sludge (DSS) via conventional extraction (CE) and ultrasound-assisted extraction (UAE). The major vibrations and local minimas obtained for our samples are highlighted in the plot for each of the sample.

Fig. 5 shows the major mid IR vibrations for the differently extracted HSs obtained using FTIR spectroscopy. Qualitative validation of functional groups such as aliphatic structure, aromatic substitution, hydrogen bond regions, and O-containing groups were analysed. Here the absolute values were plotted with Y-offset using OriginPro® (v 10.1.5.132, 2024b; OriginLab Inc., USA).

As seen in Fig. 5, C-H stretching peaks can be observed in the 2900–2800 cm^−1^ region, indicating aliphatic hydrocarbons from the HA. HA also typically shows OH stretch at 3700 cm^−1^ owing to the phenol and carboxylic groups [25]. Stronger triple bonds (e.g., −C≡C- or −C≡N) are non-typical for HA. Strong vibrational peaks around 1640 cm^−1^ are associated with the C=C stretching vibrations in aromatic compounds, such as those found in benzene rings. Additionally, this region can also indicate the presence of C=O stretch in conjugated systems like ketones, quinones and aldehydes [53], [62]. These vibrations indicated in Fig. 5 should originate from the aromatic chains of HA. This is also in line with the presence of deep UV absorption features in Fig. 3. FA extracts showed more intense vibrations around 1408 cm^−1^, indicative of a higher proportion of oxygen-containing active groups like alcohols, phenols or benzene ring structures in UAE-FA [62]. Broadened absorption peaks in the UV–Vis spectrum complement this finding. The deformation vibrations of methylene and methyl groups at 1440 cm^−1^ and 1380 cm^−1^ did have a noticeable presence in CE-HA but were intensified in the UAE. Bands around 1040 cm^−1^ are most probably attributed to Si-O stretching in silica or soil, although the presence of alcohol and alkyl ester cannot be neglected. Previous studies have indicated that the observed spectral variations are likely associated with the presence and composition of inorganic constituents [50], [53], especially based on the heterogenous nature of the feedstock. This cannot be ruled out. Overall, the ultrasound-extracted products present higher peak intensities and very similar functional fingerprints compared to the conventional extracts.

#### 3.5.4 Scanning electron microscopy analysis

**Fig. 6:**
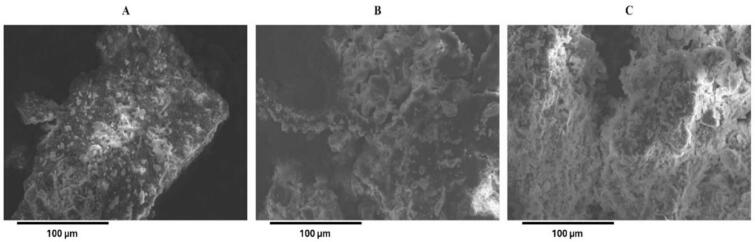
Scanning electron microscopy images of (A) dried and milled digested sewage sludge (DSS) feedstock before extraction, (B) digestate residue after conventional extraction (200 rpm, 16 h) and (C) digestate residue after ultrasound-assisted extraction (97.6%, 8.4 min). Scale bars 100 µm (magnification: 250X).

Surface microstructures of DSS feedstock and extraction residues were studied using SEM analysis. A smooth, intact texture was observed for the feedstock sample (Fig. 6.A). However, the visible physical changes observed for extraction residues (Fig. 6.B, 6.C) demonstrated the differences between ultrasound and active maceration-based methods. The use of UAE has shown to cause significant physical alterations to the surface microstructures of various materials [39]. The SEM images show that increased irregularity, surface roughness, impurity, and/or slat deposition are features of UAE residues [39] compared with CE residues. The increased mineral/salt surface adhesions are attributed to the caustic potash used in the extraction process. Fatima et al. (2021) found pre-treating feedstock chemically results in noticeable elemental compositional differences alongside inferred structural and morphological changes [45]. UAE residues portray a clear morphology departure, as cavitation-induced surface fragmentation and breakdowns led to a highly disruptive texture, increased porosity and densification on the matrix surface, and visibility seen by increased white reflective flakes (Fig. 6.C). This explains the HS recovery findings, as matrix disruption led to solvent penetration and higher HA yields.

## 4 Energy-based economic assessment

The preliminary OC analysis based on direct variable inputs, namely electrical energy and chemical reagents, estimated a total cost of 144.0 € kg^−1^ HA for UAE and 345.4 € kg^−1^ HA for CE (Table 9, S2), corresponding to a 139.8% higher cost for CE, or approximately a 2.4-fold increase relative to UAE. This difference was driven primarily by the substantially lower electrical energy demand associated with UAE, while reagent costs (KOH and HCl) were comparable between the two extraction methods due to identical chemical dosing strategies. It is also worth noting that the operational time differed markedly between the two processes, with UAE requiring approximately 8.4 min per extraction cycle, compared to 16 h for CE. While not explicitly quantified in the present assessment, this substantial reduction in processing time implies a significantly higher potential throughput for UAE at larger scales, enabling multiple extraction cycles within a single operating shift compared to batch-limited CE.

**Table 9:** Energy intensity (EI), chemical intensity (CI), and preliminary operating cost (OC) comparison for humic acid (HA) recovered.

Sl. no	Method	EI	CI	C_energy_	C_chem_	OC
		kWh g^−1^ HA	g _reagent_ g^−1^ HA	€ kg^−1^ HA	€ kg^−1^ HA	€ kg^−1^ HA
1	CE	0.277	3.054	52.615	292.784	345.40
2	UAE	0.021	1.461	3.941	140.066	144.01

CE: Conventional extraction; UAE: Ultrasound-assisted extraction; HA: Humic acids; EI: Energy intensity; CI: Total chemical (KOH + KCl) intensity; C_energy_: Energy cost; C_chem_: Chemicals cost; OC: operating cost.

Both CE and UAE simultaneously yielded FA alongside HA. In this conservative screening analysis, all energy and reagent costs were allocated exclusively to HA recovery. Accounting for FA as a co-product would further reduce the effective cost of HA production, particularly for UAE, but this was not considered here. For reference, a commercial humic acid standard from Sigma-Aldrich is priced at approximately 371.0 € kg^−1^ HA, indicating that HA recovered from digestate via UAE can be economically competitive with, and potentially favourable to, commercially available products when scaled to industrial production.

## 5 Conclusion

This study validates the effectiveness of UAE in recovering HS from real anaerobically digested sewage sludge samples. The RSM was employed to investigate and further optimise UAE extraction parameters, US amplitude (20–100%) and extraction time (5–20 min) for improving HS recovery. Extraction yields of 424.67 ± 0.03 mg HA g^−1^_db_ and 419.04 ± 0.01 mg FA g^−1^_db_ were obtained, translating to 109% increase HA in under 10 min, compared to 16-hour long CE, at optimized UAE conditions (US amplitude – 97%, extraction time 8.4 min, using 0.1 M KOH at 1:40 v/w solid to liquid ratio). Additionally, substantial drop, 85.8% (CE: 0.281 kWh vs. UAE: 0.03 – 0.04 kWh) in power consumption were also observed when utilizing optimized UAE. Spectrometric analysis revealed a notable increase in functional group diversity in the extracted HSs, with only minimal variability observed between extraction methods, despite the heterogeneous nature of the feedstock. In conjunction, the higher carbon and nitrogen contents of digestate-derived HA, relative to the commercial standard, highlight their strong potential as value-added agrochemical products. The preliminary energy and operating cost assessment further demonstrates a clear advantage of UAE. Coupled with the significantly shorter processing time, these findings indicate that UAE is well suited to high-throughput operation at larger scales. The adoption of continuous or flow-through ultrasonic reactor configurations could further facilitate scale-up while maintaining manageable operating costs. Overall, the discoveries emphasise the potential possibility of DSS, a widely available and underutilized resource, to be valorised as a sustainable feedstock for HS extraction in alignment with circular economy principles. The solid residues remaining after extraction of HS may be further directed towards downstream thermochemical valorisation using pyrolysis, or hydrothermal carbonisation, enabling the production of bioproducts such as biochar or hydrocar and bio-oil. While this study lays out foundational insights into HS, the molecular and elemental interaction studies would be needed for validating their physicochemical properties and subsequent applicability. Future studies should focus over application specific characteristics and properties to expand the understanding and utility of humic substances derived from DSS. In conclusion, this work successfully demonstrated the valorisation potential of anaerobic digestate through optimized HS recovery using UAE, contributing to sustainable waste management and resource recovery practices while reinforcing the principles of circular economy frameworks.

## CRediT authorship contribution statement

**Shon George Shiju:** Writing – original draft, Visualization, Software, Methodology, Investigation, Formal analysis, Data curation. **Krishnakumar Chullipalliyalil:** Writing – review & editing, Visualization, Resources, Formal analysis. **Brijesh K. Tiwari:** Writing – review & editing, Supervision, Resources, Formal analysis. **Patrick Quille:** Writing – review & editing, Supervision, Formal analysis. **Gaurav Rajauria:** Writing – review & editing, Supervision, Submission, Software, Resources, Funding acquisition, Conceptualization, Administration.

## Declaration of competing interest

The author declares the following financial interests/personal relationships which may be considered as potential competing interests: Shon George Shiju, Brijesh K. Tiwari, Patrick Quille and Gaurav Rajauria has patent #Ultrasound aided rapid extraction of humic substances (humic and fulvic acids) from organic wastes (digestate and sludge) as biostimulant (conformity assessment, CE mark)- Disclosure Form number IDF- 2024-006. pending to Not Licensed yet. If there are other authors, they declare that they have no known competing financial interests or personal relationships that could have appeared to influence the work reported in this paper.
